# Quality of life before and after redo IPAA: does pouch salvage improve quality of life?

**DOI:** 10.1007/s10151-025-03276-3

**Published:** 2026-03-03

**Authors:** S. D. Holubar, A. Alipouriani, O. Lavryk, J. Lipman, A. E. Kanters, B. Cohen, K. Falloon, F. Rieder, T. Qazi, E. Gorgun, S. R. Steele, D. Liska

**Affiliations:** 1 Department of Colon & Rectal Surgery, Digestive Diseases Institute, Cleveland Clinic, 9500 Euclid Ave. A30, Cleveland, OH 44195 USA; 2 Department of Gastroenterology & Hepatology, Digestive Diseases Institute, Cleveland Clinic, Cleveland, USA; 3 Cleveland Clinic Lerner College of Medicine, Case Western Reserve University, Cleveland, USA

**Keywords:** Ileoanal pouch, Pouch salvage, Quality of life, Surgery, Inflammatory bowel disease, Crohn’s disease, Ulcerative colitis

## Abstract

**Background:**

Quality of life (QoL) and functional outcomes after redo ileoanal pouch (IPAA) surgery are worse than after primary (index) pouch surgery. However, QoL in failing pouches compared to after redo IPAA has not been reported. We hypothesized that QoL after redo IPAA would be improved compared with failing IPAAs immediately prior to redo surgery.

**Methods:**

Adults who underwent redo IPAA (1984–2024), had successful loop ileostomy closure, and at least one pouch survey were included. Our primary outcome was the Cleveland Global Quality of Life Index (CGQLI; range, 0 [lowest] to 1 [highest]). Survey responses before and after redo IPAA were compared using unmatched and matched pair analyses.

**Results:**

A total of 528 redo pouches were included: pouch excision with neo-IPAA in 318 (60%) and pouch repair with neo-IPAA in 210 (40%); 298 (56%) had follow-up survey data available. After redo IPAA, social, work, and sexual restrictions all decreased (*p* ≤ 0.05), and bowel movements decreased from 10 to 8 per 24 h (*p* = 0.05), whereas urgency, incontinence, seepage, pad usage, fiber, antidiarrheal usage, and dietary restrictions were comparable to before redo IPAA. After redo IPAA, quality of health, quality of energy, QoL, and CGQLI were significantly higher than those before redo IPAA (*p* < 0.0001). When surveyed after redo IPAA, 86% of patients would undergo redo surgery again, 88% would recommend redo IPAA, and happiness with surgery increased (*p* = 0.001).

**Conclusion:**

Redo IPAA improved quality of life and restrictions observed prior to redo pouch surgery without compromising functional outcomes for most patients.

## Introduction

Ileal pouch-anal anastomosis (IPAA) is the surgical procedure of choice for patients with ulcerative colitis (UC) and familial adenomatous polyposis (FAP) requiring proctocolectomy, offering the opportunity for maintenance of transanal defecation, avoidance of a permanent stoma, and essentially normal quality of life (QoL) [1, 2]. Despite its success, approximately 10–15% of patients experience complications that may result in poor QoL and pouch failure, and some may benefit from pouch salvage with a redo IPAA to avoid a permanent ileostomy [3, 4].

Compared with primary IPAA, redo IPAA is associated with a lower success rate (75% vs. > 90% with primary IPAA) and a higher complication rate. Patients undergoing redo procedures report worse outcomes in terms of QoL and functional outcomes compared to primary IPAA—in part related to the higher proportion of a handsewn anastomosis frequently required in redo IPAA, and foreshortening of the length of small bowel left in situ if the old pouch needs to be excised [3, 5].

However, there are no reports of patient-reported outcomes (PROs) after redo IPAA compared with those experiencing pouch failure prior to redo IPAA to determine whether PROs improve after pouch salvage. PROs are crucial for understanding the real-world impacts of surgical interventions and provide insight into the subjective patient experience [6]. This study aimed to determine the impact of redo IPAA on QoL, functional bowel symptoms, and adaptive aspects of patients with pouch failure. We hypothesized that redo IPAA is associated with improved QoL compared with QoL before redo IPAA.

## Methods

Our pouch registry was used to identify adult (age ≥ 18 years) patients who had initially undergone primary (index) IPAA at our center or elsewhere and subsequently underwent redo IPAA at our center. Redo IPAA procedures included pouch excision with reconstruction of a new pouch (neo-IPAA) or repair of the existing pouch with re-anastomosis. Inclusion criteria included ≥ 3 month follow-up and available survey data and included the latest survey prior to redo IPAA and/or the most recent survey after loop ileostomy closure after redo IPAA. To allow for a meaningful analysis of PROs after redo IPAA, patients without documented diverting loop ileostomy closure were excluded.

### Pouch surveys

The Cleveland Clinic Pouch Survey is a validated measure of various PRO metrics (Table 1), including bowel frequency, urgency, incontinence, seepage, pad use, and medication use. Pouch surveys were mailed annually to patients and were also administered in person during clinic visits. In addition, several items (restrictions, overall QoL, and incontinence scores) were administered digitally prior to clinic visits between 2015 to 2025. Survey responses before and after redo IPAA were compared using chi-squared or Wilcoxon rank-sum test for unpaired data. For the subset of patients with both before and after redo IPAA survey data, a matched-paired sensitivity analysis with paired *t* tests or McNemar’s tests was performed.

**Table 1:** Pouch survey items

Domain	Item	Answer choices
Bowel frequency	Bowel movements/day	Numeric
	Bowel movements/night	Numeric
	Bowel movements/24 h	Numeric
	Stool frequency	Usual stool frequency since pouch 1–2 stools more/day more than usual 3 or more stools more/day more than usual
	Bristol Stool Score	1 (hard lumps) to 7 (watery, no solid pieces)
Symptoms	Abdominal pain	None vs. mild vs. moderate vs. severe
	Abdominal bloating	None vs. mild vs. moderate vs. severe
	Abdominal cramping	None vs. mild vs. moderate vs. severe
	Fecal urgency*	Never vs. rarely vs. sometimes vs. mostly vs. always
	Fecal incontinence**	Never vs. rarely vs. sometimes vs. mostly vs. always
	Fecal Incontinence Severity Index	0 (best)–61 (worst)
	Wexner score	0–20 (worst)
	Fecal seepage, day?	Yes/no
	Fecal seepage, night?	Yes/no
	Wear pads for seepage, day?	Yes/no
	Wear pads for seepage, night?	Yes/no
	Pad reason?	None vs. peace of mind vs. necessity
Medications	Antidiarrheals?	Yes/no
	Antibiotics?	Yes/no
	Steroids?	Yes/no
	Fiber?	Yes/no
	Anti-IBD medications?	Yes/no
Restrictions	Social	Yes/no
	Work	Yes/no
	Dietary	Yes/no
	Sexual	Yes/no
Quality of life measures	Quality of life (0–10)	0 (worst)–10 (best)
	Quality of health	0 (worst)–10 (best)
	Energy level	0 (worst)–10 (best)
Satisfaction	Would you have pouch surgery again?	Yes/no
	Recommend surgery to someone else?	Yes/no
	Satisfaction with results of surgery?	0 (worst)–10 (best)

*IBD *inflammatory bowel disease

*Inability to defer bowel movements for more than 30 min

**Accidents of bowel movements

### Primary outcome

The primary outcome of interest was the Cleveland Global Quality of Life Index (CGQLI), a validated composite measure of overall QoL, including health status and energy levels [7–9]. The CGQLI includes QoL, quality of health (QoH), and quality of energy (QoE). Each component was scored from 0 (lowest) to 10 (highest), and the CGQLI was calculated by summing the three scores and dividing by 30, yielding an overall score ranging from 0 (lowest) to 1 (highest).

## Results

### Patient demographics

**Fig. 1:**
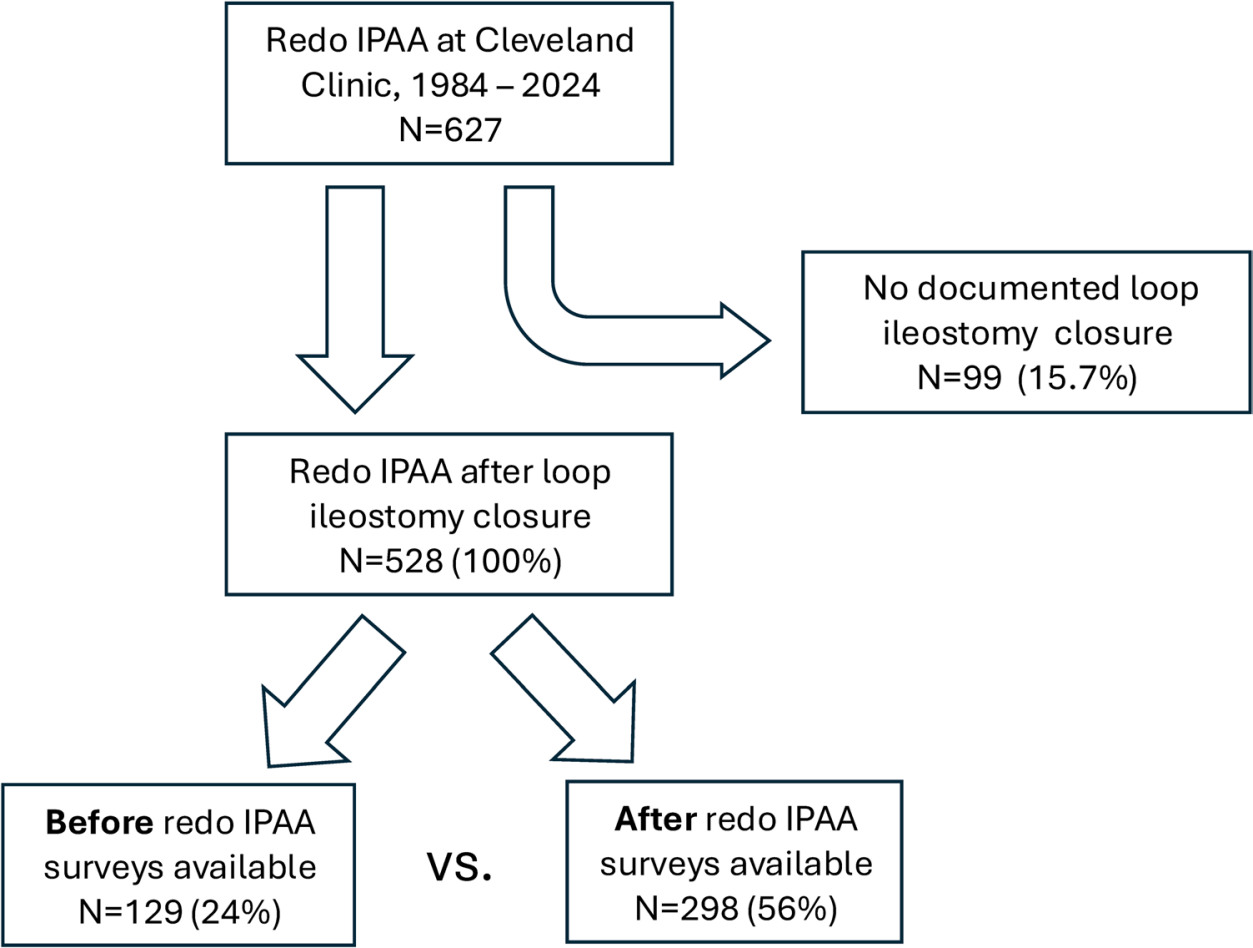
CONSORT-style diagram of analyzed patients

We identified 627 adult redo IPAA procedures performed by 36 surgeons (median 4, interquartile range [IQR] 2–16) at our institution between 1984 and 2024, during which time 5430 index pouches were performed. Of these, 99 (15.7%) had no documented diverting loop ileostomy closure and were excluded, leaving 528 patients for analysis (Fig. 1). The baseline characteristics at the time of redo IPAA are presented in Table 2. The median age was 38 years, 58% were women, and the median BMI was 23.4 kg/m^2^. Of the 528 patients, 318 (60%) underwent pouch excision with neo-IPAA and 210 (40%) underwent pouch repair with neo-IPAA. The median (IQR) time from redo pouch to stoma closure was 3.8 months (3.2–5.7 months).

**Table 2:** Baseline characteristics at the time of redo IPAA in patients who underwent ileostomy closure

Variable	*N* = 528
Age	38 (28–48)
Gender, female	306 (58%)
BMI, kg/m^2^	23.4 (20.6–27.1)
ASA 3 or 4	125 (23.6%)
Diagnosis	
UC	458 (86.7%)
FAP	32 (6.1%)
CD	30 (5.7%)
Other	8 (1.5%)
Type of surgery	
Pouch excisions with neo-IPAA	318 (60%)
Pouch repairs with neo-IPAA	210 (40%)
Handsewn anastomosis	393 (74.4%)
Diverting loop ileostomy	528 (100%)
Duration of surgery, hours	4.7 (3.9–5.6)
Estimated blood loss, mL	300 (150–381.2)
Time to ileostomy reversal, months	3.8 (3.2–5.7)

The figures represent the median (interquartile range) or frequency (proportion). *P* values represent chi-squared or Wilcoxon rank-sum tests

*ASA* American Anesthesiology Classification, *BMI* body mass index,* CD* Crohn's disease,* FAP* familial adenomatous polyposis, *IPAA* ileal pouch-anal anastomosis,* UC* ulcerative colitis

Of the 528 patients who underwent redo pouch procedure followed by stoma closure; of these, 129/528 (24%) had baseline (before redo IPAA) survey data available, and the median time from baseline survey to redo pouch was 7 (IQR 2–14.5) months. After redo pouch followed by stoma closure, 298/528 (56%) patients had follow-up survey data available, and the median time from redo pouch to the follow-up surveys was 27.5 (IQR 13–61) months.

### Patient-reported outcomes

The functional outcomes are presented in Table 3. After redo IPAA, compared with before redo IPAA, the median number of bowel movements per 24 h decreased from 10 to 8 (*p* = 0.05; matched pair *p* value 0.31), and there was no significant difference in other functional outcomes, including seepage, urgency, and incontinence.

**Table 3:** Bowel function and symptoms before and after redo IPAA

Variable	*N* before vs. after redo	Before redo pouch	After redo pouch	Delta/interpretation	*p* value**	Matched pairs	*p* value***
Redo IPAA to survey, months	129 vs. 298	7 (2–14.5)	27.5 (13 to 61)		–	65	–
Bristol stool score	61 vs. 51	6 (6–7)	6 (5–7)	Same	0.39	8	0.76
Bowel frequency							
Daytime	80 vs. 270	7 (5.25–8)	6 (5–8)	1 less	0.27	24	0.33
Nighttime	91 vs. 271	3 (2–4)	2 (1–3)	1 less	0.002^a^	26	0.66
Total	80 vs. 270	10 (7–12)	8 (6–11)	2 less	0.05^a^	23	0.31
Seepage							
Daytime	36 vs. 188	50 (55%)	93 (49%)	6% less	0.50	13	0.62
Nighttime	35 vs. 189	27 (77%)	130 (68.8%)	8% less	0.32	15	1.0
Abdominal*							
Cramping	41 vs. 189	12 (29.3%)	42 (22.2%)	7% less	0.33	21	1.0
Bloating	40 vs. 188	11 (27.5%)	44 (23.4%)	4% less	0.58	20	1.0
Pain	41 vs. 191	9 (22%)	42 (21.9%)	same	1.0	21	1.0
Fecal urgency	124 vs. 203	93 (75%)	140 (69%)	6% less	0.24	15	1.0
Fecal incontinence	97 vs. 79	26 (26.8%)	31 (39.2%)	13% more	0.08	16	1.0
Incontinence scores							
FISI: 0–61 (worst)	84 vs. 59	20.5 (0–38)	28 (12–39)	8 points worse	0.1	19	0.15
Wexner: 0–20 (worst)	54 vs. 42	13.5 (9–16)	14 (11–16.25)	0.5 points worse	0.29		

The figures represent the median (interquartile range) or frequency (proportion)

*FISI* Fecal Incontinence Severity Index,* IPAA* ileal pouch-anal anastomosis

*Moderate-to-severe

**Chi-squared or Wilcoxon rank-sum test

***Matched paired* t* test or McNemar’s test as appropriate

^a^Significant (*p* ≤ 0.05)

**Fig. 2:**
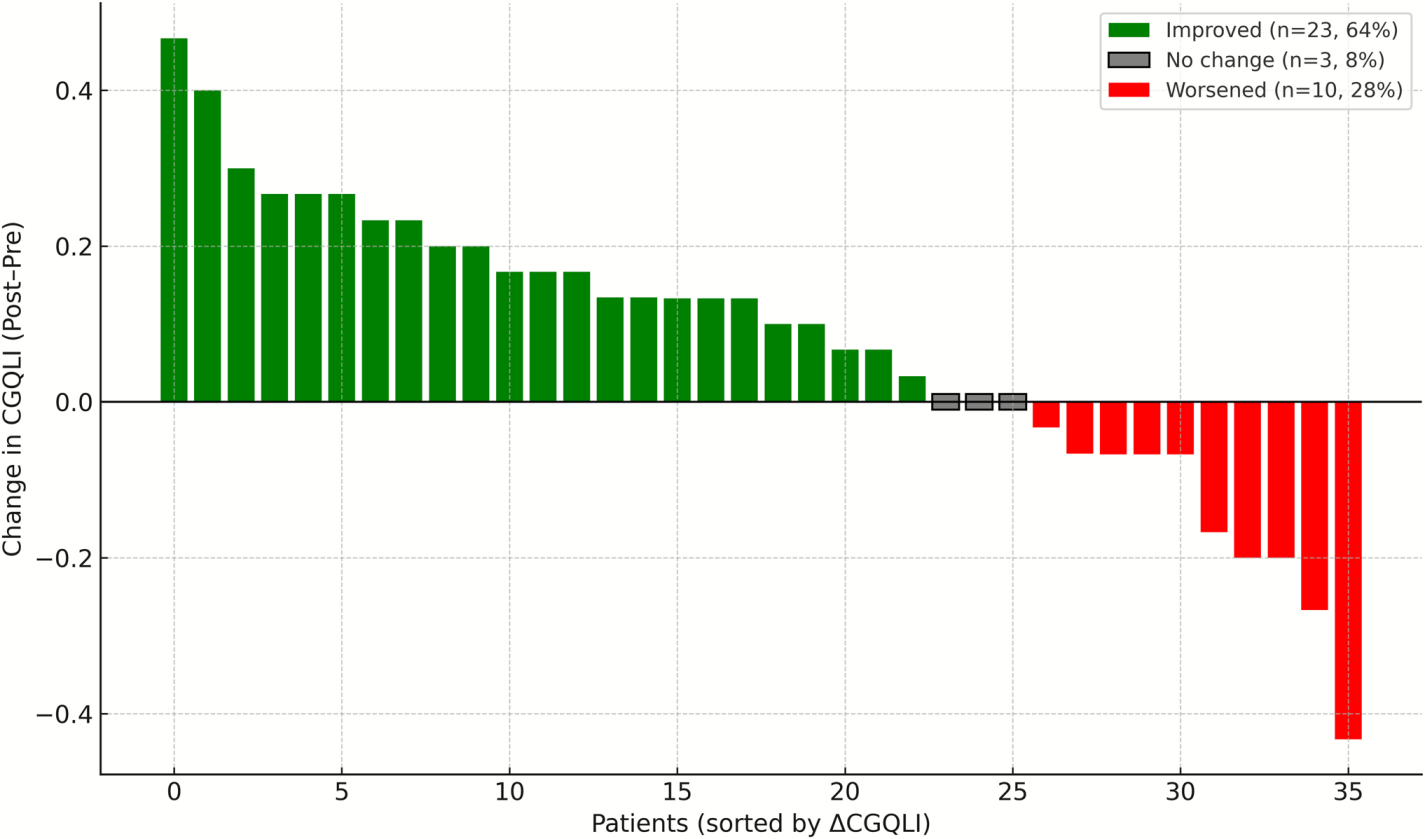
Waterfall plot of individual changes in CGQLI after redo IPAA (matched pairs, *n* = 36)

Patient adaptations, restrictions, QoL, and satisfaction with surgery are presented in Table 4. Overall, there was no significant difference in the use of pads, fiber, and antidiarrheal medications or dietary restrictions after redo IPAA compared with before redo IPAA, whereas social, work, and sexual restrictions all decreased after redo IPAA (*p* < 0.05). All three QoL items (QoL [from 7 to 8], quality of health [from 6 to 8], and quality of energy [from 6 to 7]) increased from before to after redo IPAA (*p* < 0.0001; matched pair *p* < 0.03), as did the CGQLI, from 0.57 to 0.73 after redo IPAA (*p* < 0.0001, matched pair *p* = 0.002). Among 36 matched pairs, 64% improved, 8% were unchanged, and 28% worsened in CGQLI after redo IPAA (Fig. 2). In terms of satisfaction with surgery after redo IPAA, compared with responses before redo IPAA, a slightly lower proportion of patients reported that they would undergo redo IPAA again (91.7% vs. 85.9%) or would recommend it IPAA to friends or family members (91% vs. 88.2%). The median satisfaction rating with surgery increased from 8 to 9 (*p* = 0.001; matched *p* value 0.02) before and after redo IPAA, but these differences were not significant (all *p* > 0.05).

**Table 4:** Adaptation, restrictions, satisfaction, and quality of life before and after redo IPAA

Variable	*N* before vs. after redo	Before redo pouch	After redo pouch	Delta/interpretation	*p* value*	Matched pairs	*p* value**
Redo IPAA to survey, months	218 vs. 172	− 1 (− 0 to − 6)	18 (9–52)	**	–	–	–
Pad usage							
Daytime	53 vs. 207	23 (43%)	108 (52%)	9% more	0.25	24	1.0
Nighttime	53 vs. 208	33 (62%)	163 (64%)	2% more	0.82	25	1.0
Reason: necessity	29 vs. 164	17 (58.6%)	164 (50%)	8% less	0.30	11	1.0
Medications							
Antidiarrheal	32 vs. 172	99 (57.6%)	120 (56%)	1% less	0.25	16	1.0
Antibiotics	27 vs. 155	5 (18.5%)	28 (18.1%)	Same	0.95	12	1.0
Steroids	28 vs. 152	4 (14.3%)	6 (3.9%)	10% less	0.03^a^	14	0.48
Fiber	25 vs. 190	5 (20%)	38 (24.4%)	4% more	0.63	11	0.48
Anti-IBD	23 vs. 146	2 (8.7%)	5 (3.4%)	5% less	0.24	10	1.0
Restrictions							
Dietary	217 vs. 164	109 (50.2%)	80 (48.8%)	2% less	0.78	37	0.54
Social	216 vs. 158	90 (41.7%)	50 (31.6%)	10% less	0.05^a^	38	0.55
Work	217 vs. 155	109 (50.2%)	54 (34.8%)	15% less	0.003^a^	38	0.77
Sexual	212 vs. 160	84 (39.6%)	43 (26.9%)	11% less	0.01^a^	36	0.18
QoL items: 0–10 (best)							
Quality of health	212 vs. 160	6 (4–8)	8 (6–9)	20% better	< 0.0001^a^	36	0.03^a^
Quality of energy	215 vs. 161	6 (4–7)	7 (5–8)	10% better	< 0.0001^a^	36	0.0007^a^
Quality of life	212 vs. 168	6 (4–8)	8 (6–9)	20% better	< 0.0001^a^	37	0.003^a^
CGQoL: 0–1 (best)	204 vs. 150	0.57 (0.4–0.73)	0.73 (0.6–0.83)	16% better	< 0.0001^a^	36	0.002^a^
Satisfaction							
Undergo surgery again	48 vs. 199	44 (91.7%)	171 (85.9%)	5% fewer	0.28	26	0.68
Recommend surgery	44 vs. 187	40 (91%)	165 (88.2%)	3% fewer	0.61	22	0.62
Satisfaction with surgery: 0–10 (best)	47 vs. 205	8 (5–9)	9 (7–10)	1 point higher	0.001^a^	26	0.02^a^

The figures represent the median (interquartile range) or frequency (proportion)

*IBD *inflammatory bowel disease, *IPAA* ileal pouch-anal anastomosis, *CGQLI* Cleveland Global Quality of Life Index, *QoL* quality of life

*P values represent Wilcoxon rank-sum test

**Matched paired *t* test or McNemar’s test as appropriate

^a^Significant (*p* ≤ 0.05)

## Discussion

Our findings indicate that patients who develop pouch failure before undergoing redo IPAA experience decreased QoL, and redo IPAA results in significant improvements in these measures without compromising functional outcomes in most patients on both unmatched and matched paired analyses. Additionally, after redo IPAA, 86% of patients were willing to undergo IPAA again, and 88% were willing to recommend IPAA, highlighting the perceived benefits of this procedure in patients facing pouch failure.

The clinical presentation of complications leading to redo IPAA may manifest as increased bowel-related symptoms. These complications are associated with significantly impaired QoL, highlighting the need for salvage interventions, such as redo IPAA, to improve patient health. Our study found that while urgency, incontinence, and other functional parameters remained largely unchanged, QoL indicators showed significant improvement. The lack of difference in functional outcomes is likely attributable to the higher rate of pouch excision with neo-IPAA (60%) and the higher rate of handsewn anastomoses (74.4%) compared with index IPAA, which may result in increased stool frequency and seepage, respectively. This suggests a trade-off, with patients accepting a lack of improvement in functional outcomes for their ability to be restored to health without a permanent ileostomy. Overall, social, work, and sexual restrictions were lower after redo IPAA, indicating that patients were more functional individuals, which is a critical aspect of the overall QoL for patients with chronic health issues.

Despite the inherent challenges associated with redo IPAA, such as increased risks compared with primary IPAA, our data show that patients generally experience improved postoperative well-being and satisfaction. The lack of decline in patient-reported satisfaction scores, along with the significant improvement in the CGQLI, highlights the restorative potential of redo IPAA in patients with failed primary pouches. The willingness of a high percentage of patients to recommend redo IPAA and undergo the procedure again further supports the positive impact of this surgical intervention. These patients valued the ability to avoid permanent ileostomy, which may have played a significant role in their reported satisfaction. These findings are consistent with previous studies that emphasize the psychological and emotional benefits of maintaining continence and avoiding a permanent stoma [10–12].

This study has several limitations. The retrospective nature of data collection may introduce selection bias, as patients who responded to the surveys may have had different experiences than those who did not respond. Additionally, reliance on self-reported data can lead to recall bias and subjectivity. The changing methods of survey administration over the years led to inconsistent response rates, and a relatively small number of patients with paired pre- and post-redo IPAA surveys. As such, the study was underpowered for subgroup comparisons and did not stratify by indication, surgical technique (such as handsewn), or complications. Given the focus on overall quality of life after redo IPAA, our intent was to capture the average patient QoL after redo IPAA. In addition, our study lacks a comparator; ideally, these PRO measures would be assessed relative to patients who underwent pouch excision in lieu of redo IPAA. Finally, as a quaternary referral center, this cohort reflects a select group of highly motivated patients with a strong preference to avoid a permanent ileostomy and thus pursue pouch salvage, which may limit generalizability. Despite these limitations, the large sample size and long-term follow-up provide unique insights into the outcomes of redo IPAA from a patient perspective.

In summary, redo IPAA improved overall quality of health and life without negatively affecting bowel function or increasing other functional impairments in most patients. These findings provide valuable insights into what patients may reasonably expect after redo IPAA and underscore the importance of considering redo surgery as a valid alternative to pouch excision and permanent ileostomy for patients facing pouch failure.

## Data Availability

The data generated during this study are available from the corresponding author upon reasonable request.
